# Circulating Cell-free DNA Fragmentomics Detection and Beyond

**DOI:** 10.14336/AD.2025.1029

**Published:** 2026-09-03

**Authors:** Tianliang Liu, Zhicheng Li, Shifu Chen, Jiasheng Zhong

**Affiliations:** ^1^LifeX Institute, Gannan Medical University, Ganzhou, China; ^2^Shenzhen Institute of Advanced Technology, Chinese Academy of Sciences, Shenzhen, China; ^3^HaploX Biotechnology, Shenzhen, China

**Keywords:** Cell-free DNA (cfDNA), cancer biomarkers, cfDNA fragmentomics, fragmentomics review

## Abstract

Circulating cell-free DNA (cfDNA) comprises DNA fragments released into bodily fluids via apoptosis, necrosis, or phagocytosis. cfDNA encapsulates both fragmentomics (structural) and non-fragmentomics (sequence/epigenetic) information from source cells, thereby representing a promising biomarker. While non-fragmentomics analyses have enabled diverse diagnostic applications, they often falter in diseases with subtle or widespread genomic changes due to low cfDNA abundance and clonal hematopoiesis interference. Emerging evidence reveals that cfDNA fragmentation is shaped by nucleosome occupancy, nuclease activity, and epigenetic factors, yielding distinct patterns in fragment size, end motifs, nucleosome footprints, and topology. These fragmentomics signatures diverge markedly between healthy and diseased states, and across age group, offering opportunities to complement non-fragmentomics and enhance accuracy. This review delineates key cfDNA fragmentomics targets, elucidates fragmentation mechanisms, and explores clinical applications in the context of diseases and aging. We further survey cutting-edge technologies and computational algorithms and discuss implementation challenges alongside future prospects.

## Introduction

The role of cell-free DNA (cfDNA) in disease research and application has been extensively discussed, with numerous products currently undergoing clinical testing or application stages [1-3]. As a specific and sensitive biomarker, cfDNA can be utilized for disease detection, treatment efficacy monitoring, and providing sensitive, long-term prognostic information post-treatment or surgery [4, 5]. The non-invasive, continuous, and high-sensitivity nature of cfDNA testing offers a significant advantage in monitoring disease development over conventional methods like imaging scanning or tissue biopsy.

**Figure 1.**
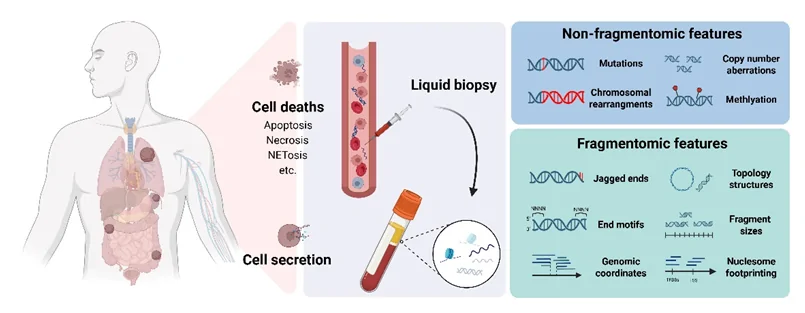
**Molecular features of cfDNA**. Schematic overview of cfDNA characteristics, categorized into non-fragmentomics features (e.g., mutations, copy number aberrations, chromosomal rearrangements, and methylation) and fragmentomics features (e.g., jagged ends, end motifs, fragment sizes, genomic coordinates, nucleosome footprints, and higher-order topologies).

Despite the promises and potential, current methodologies primarily focus on non-fragmentomics related to genetic and epigenetic alterations. However, these detections face significant challenges due to the limited quantity of cfDNA variants in bodily fluids, which varies considerably depending on the disease stage and typically corresponds to only 1500 to 3000 copies of the haploid human genome [6, 7]. Detecting variants from such trace amounts of cfDNA outweighs existing technological capabilities. A standard solution is to refine the detection threshold, but it increases false positives and negatives inevitably [8]. Nevertheless, clonal hematopoiesis complicates the interpretation of cfDNA variants [9-11]. As exemplified by epithelial cancers, many of them do not exhibit a clear carcinogenesis landscape [12, 13]. The term "cfDNA fragmentomics" originated in a 2012 patent by Franck et al., which highlighted shorter cfDNA fragments in cancer patients, enabling differentiation from healthy individuals via qPCR. In 2015, Ivanov et al. linked cfDNA fragmentation to nucleosome occupancy and epigenetic patterns [14]. Subsequently, Cristiano et al. conducted the first proof-of-principle study across multiple cancers, demonstrating that fragmentation profiles could pinpoint subtypes and tissue origins [15]. Interest has since surged, expanding from basic traits like fragment length and end motifs to intricate aspects such as topology [16]. As illustrated in Figure 1, cfDNA includes non-fragmentomics features (eg., point mutations, copy number aberrations, chromosomal rearrangements, and methylation) and fragmentomics features (eg., jagged ends, end motifs, fragment size distribution, genomic coordinates, nucleosome footprints, and higher-order topologies). These features reflect biological processes of cfDNA release, including apoptosis, necrosis, NETosis, and active secretion, and provide complementary information for disease detection. Methods and algorithms for analyzing each feature are described in subsequent sections.

## The Size of cfDNA and Characterizing Methods

**Figure 2.**
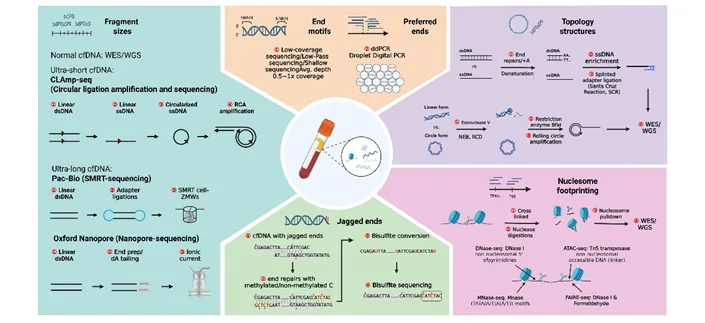
**Overview of cfDNA fragmentomics features and characterization methods**. Schematic summary of major cfDNA fragmentomics signatures including fragment size distributions, end motifs and jagged ends, topological structures, and nucleosome footprints and representative sequencing or enrichment strategies used for their detection.

cfDNA in the circulating system is highly fragmented, with an observed maximum 166 bp and a second peak at 143 bp, or multiples thereof. The size distribution aligns with DNA molecules (146 bp) wrapped around a mono-nucleosome unit plus a 20 bp linker DNA bound to histone H1 [17]. Another notable characteristic is the 10 bp periodicity between small cfDNA molecules, which allows the DNA helix to interact with the nucleosome core in 10 bp per turn. Earlier studies have utilized these characteristics to isolate circulating tumor and fetal-free DNA [18, 19]. This is primarily important because the length of these cfDNA are typically shorter than that from normal cells [20-22]. The precise mechanism underlying this phenomenon remains unclear. However, evidence indicates that the timing of nuclease cleavage may have substantial effects on cfDNA characteristics [23, 24]. Additionally, epigenomic processes may contribute, as hypomethylated cfDNA fragments are generally shorter than hypermethylated fragments [25, 26]. As summarized in Figure 2, cfDNA fragmentomics can be characterized with complementary approaches. Size profiling is performed with CLAmp-seq for ultra-short cfDNA (<150 bp) and with SMRT or Nanopore sequencing for ultra-long molecules (>600 bp). End motifs and jagged ends are detected with LC/LP-sequencing combined with ddPCR or with modified bisulfite sequencing, reflecting nuclease activity and cleavage patterns. Topological structures, including circular and single-stranded forms, are identified through enrichment protocols, whereas nucleosome footprinting reveals chromatin accessibility and tissue of origin. Together, these methods provide a multidimensional view of cfDNA fragmentation and its biological and clinical significance.

One of the most promising applications of cfDNA size profiling is noninvasive prenatal testing (NIPT). Yu et al. showed that trisomic fetuses display altered cfDNA fragment lengths on affected chromosomes, enabling trisomy 21 and 18 detection with 100% sensitivity and specificity [27]. Size-based algorithms also facilitate fetal de novo mutation detection, overcoming prior challenges [40]. In oncology, Jiang et al. reported enriched short fragments in hepatocellular carcinoma (HCC) [21], while Mouliere et al. also noted elevated short fragments in cfDNA-abundant cancers like lung and colorectal [41]. Notably, fragmentation extent correlates spatiotemporally with SNVs and CNVs [15], underscoring its diagnostic synergy (Table 1).

The apparent size of cfDNA can be significantly influenced by pre-analytical factors, such as sample preservation, DNA isolation and library preparation. Common cfDNA detections typically rely on conventional bridge amplification and NGS, which favor DNA molecules ranging from 100 to 500 bp in length. These biases primarily result in the recovery of cfDNA as double-stranded DNA (dsDNA) around 166 bp. However, more recent adoption of CLAmp-seq, SMRT-seq and Nanopore sequencing, approaches that specific for single-stranded or long DNA molecules, yield two distinct populations namely “ultra-short” and “ultra-long” cfDNA, respectively [42, 43]. Mouliere et al. observed a reduction in ultra-short cfDNA fragments are often enriched at transcription start sites (TSS) in cancer patients, suggesting that these fragments might play a regulatory role in gene expression [44-47]. However, much less is known about the biological properties of ultra-long cfDNA fragments. A notable study associated with ultra-long cfDNA is conducted by Lo et al., they profile cfDNA in pregnancies and found that average 20% of cfDNA in maternal plasma indeed exceeds 600 bp, with the longest fragment observed reaching 23,635 bp. Interestingly, they observed a significant reduction in ultra-long cfDNA in pregnancies with preeclampsia compared to those without, suggesting that ultra-long cfDNA could serve as a novel biomarker for pregnancy-associated disorders.

**Table 1:** Fragmentomics-based assay and applications.

Features	Analytical population	Topic/Application	Methods	Key results	Reference
**Fragment sizes**	Ultra-short cfDNA	Early cancer detection of multiple cancer types	CLAmp-seq	Cancer patients exhibited a higher proportion of ultra-short cfDNA fragments compared to healthy individuals. The coverage of CTCF binding sites was significantly reduced.	Chen et al [28].
	Ultra-short cfDNA	Predicting early recurrence of biliary tract cancer	High-sensitivity DNA chip	Patients in the early recurrence (ER) group showed a significantly higher proportion of ultra-short cfDNA fragments (31.0% vs. 15.7%, p < 0.001) compared to the non-ER group	Kim et al [29].
	Ultra-long cfDNA	Predicting response to the immune-checkpoint inhibition (ICI) and progression of pan-cancer patients	Modified ADELIS	Patients with higher quantity of fragments over 1650 bp were likely not to progress with better response to ICI.	Benzekry et al [30].
**End signatures**	End motifs	Hepatocellular carcinoma, colorectal cancer, non-small cell lung cancer, and esophageal carcinoma	Archived databases analysis	Certain end motifs (“TATT”, “CGTC”) are preferentially apparent in cancer group.	Li et al [31].
	Preferred ends	Fetal trisomy 21 testing	Whole genome bisulfite sequencing	Short-fragment preferred ends are enriched in fetal DNA, driven by preferential cleavage at nucleosome cores.	Lo et al [32].
	Jagged ends	Familial systemic lupus erythematosus (DNASE1l3 deficiency) diagnosis	Jag-sequencing	Reduced jaggedness is strongly associated with familial SLE caused by DNASE1L3 dysfunction, which facilitates impaired chromatin digestion and dysregulated cfDNA release.	Lo et al [33].
**Topology structures**	Single-stranded	Lung cancer detection	ssDNA enrichment-assisted WGS	Tumor patients exhibit elevated proportions of single-stranded cfDNA, likely attributable to heightened genomic instability and dysregulated DNA release mechanisms (e.g., apoptosis, NETosis, or active secretion.	Su et al [34].
	Circular extrachromosomal DNA (ecDNA)	Monitor of oncogenes dosage	Circular sequencing	ecDNA copy number drives MYC dosage heterogeneity in pancreatic ductal adenocarcinoma.	Corbo et al [35].
	Extrachromosomal rDNA (ERC)	DNA damages and aging indicators	N/A	Collisions between transcription and stalled replication forks drive rDNA instability, leading to copy number alterations and age-associated accumulation of ERCs.	Kobayashi et al [36].
	MicroDNA	Monitor of cancer progression and therapeutic efficacy	DNASE-assisted WGS	Post-surgery decrease in microDNA length correlates with tumor burden reduction.	Dutta et al [37].
**Nucleosome footprinting**	N/A	Detecting nucleosome reorganization and gene regulatory patterns in breast cancer	MNase-assisted histone H3 ChIP-seq	Tumor tissues exhibit a 5-10 bp decrease in nucleosome repeat length, resembling pluripotent stem cells.	Vergote et al [38].
	N/A	Molecular subtypes classification of prostate cancer	ATAC-seq	Androgen receptor-active tumor shows nucleosome depletion at androgen receptor (AR) binding sites, while neuroendocrine subtype exhibit depletion at ASCL1 binding sites.	Ha et al [39].

This table illustrates the diverse fragmentomics-based assays currently employed to analyze cfDNA characteristics and their clinical applications across various disease states. However, direct comparison of assay performance across studies is challenging due to inconsistent reporting of modeling approaches, variable sample selection criteria, and lack of standardized performance metrics. Additionally, many studies may contain inherent biases from their carefully selected sample cohorts. ER, early recurrence; ICI, immune-checkpoint inhibition; ecDNA, extrachromosomal DNA; ERC, extrachromosomal rDNA; AR, androgen receptor.

The size of cfDNA is typically determined using conventional NGS methods, unless the specific populations mentioned above are the focus of the analysis. For these purposes, approaches such as shallow whole genome sequencing (sWGS), low-pass whole genome sequencing (LP-WGS) and low-coverage whole genome sequencing (LC-WGS) are often employed. In specific, sWGS typically uses ~0.1-1× coverage for broad genomic surveys, LP-WGS employs ~1-5× coverage for cost-efficient profiling, and LC-WGS, often <1×, is optimized for fragment analysis, as seen in the DELFI method for ultra-short cfDNA [15].These methods allow for position-specific adaptation to increase resolution and coverage. For example, Cristiano et al. used DELFI, an adapted LC-WGS technique, to compare cfDNA fragment lengths in cancer patients, analyzing them within 5 Mb bins [15]. In addition to these technical advancements, machine learning (ML) and deep learning (DL) algorithms are being applied to cfDNA size profiling, ranging from simple logistic regression to more complex multilayer artificial neural networks [48].

## The End signatures of cfDNA and Characterizing Methods

Due to sequencing resolution limitations and the paired-end repair step during library preparation, the end characteristics of cfDNA had not been widely studied. It wasn't until cfDNA sequences were analyzed at base resolution that Chan et al. identified a subset of genomic coordinates preferentially cleaved during the generation of plasma cfDNA, which they termed "preferred ends" [49]. It is now clear that at least two key mechanisms contribute to the formation of these preferred ends. First, the genome tends to cleave at certain accessible regions. Second, cfDNA cleavage is mediated by different nucleases, each with unique cutting preferences [40, 49, 50]. Based on these contexts, it can be further categorized into end coordinates, 5'-end motifs, and single-stranded 5' or 3' protruding ("jagged") ends.

Chan et al. were the first to demonstrate that fetal cfDNA exhibits a specific 4-mer end [49]. Building on this, Sun et al. found that fetal cfDNA is predominantly cleaved within nucleosome cores, while maternal cfDNA is primarily cleaved at linker regions, resulting in distinct end sequences [32]. Similarly, Jiang et al. analyzed cfDNA from liver transplant recipients and HCC patients, identifying two independent end coordinates specific to each group [51]. These findings suggest that cfDNA end patterns vary depending on the physiological conditions, highlighting the potential of end sequences as informative biomarkers. Supporting this, Chandrananda et al. observed a prevalence of C-ends in autosomal cfDNA, a feature not seen in cfDNA from other sources [52]. Serpas et al. linked this to DNASE1L3 inactivation, as they found reduced DNASE1L3 activity significantly diminishes the 5' end predominance in cfDNA fragments [53]. Notably, lower DNASE1L3 expression is commonly observed in cancer patients, which may help explain the altered end sequences in these individuals [54-56]. Besides, at least two other DNA nucleases, DFFB and DNASE1, have been shown to play roles as well. DFFB prefers creating A-ends, while DNASE1 favors nucleosome-free cfDNA, producing fragments with T-ends [50, 57]. Furthermore, over 80% of cfDNA fragments exhibit single-stranded DNA ends, known as jagged ends [58]. Jaggedness is more pronounced in fetal cfDNA compared to maternal samples and is also observed in samples from patients with bladder cancers or urinary infections [58-65].

Characterizing cfDNA end signatures remains a significant challenge, primarily due to the complexity and heterogeneity of cfDNA fragments. Traditional methods used to validate DNA breakpoints, such as gel electrophoresis, comet assays, in situ ligation, PCR, and TUNEL assays, are not suitable for profiling cfDNA ends. These methods are generally designed to detect the presence of DNA breaks and are not sensitive enough to distinguish subtle variations of the DNA ends. Alternatively, more recent methods, like BLISS (Breaks Labeling In Situ and Sequencing) and End-Seq, have been developed to address these limitations and are now widely applied in cfDNA analysis [66, 67]. And a recent variation, known as "Jag-seq", incorporates methylation signals during the end-repair process. It leverages the fact that CpG dinucleotides in the genome are highly methylated in a tissue-specific manner and preserved in cfDNA [61]. Xie et al. have further refined this method by using methylated cytosines instead, claiming that this modification enhances resolution [68]. Nevertheless, it is important to consider experimental conditions such as sonication and the use of reagents like heparin or EDTA, as these may alter the cfDNA end signatures [68].

Despite rapid methodological advances, cfDNA fragmentomics remains highly susceptible to pre-analytical bias, and field-wide standards are still maturing. Blood collection tube chemistry and time-to-plasma isolation alter both cfDNA yield and inferred tissue contributions and can reshape size distributions relevant to fragmentomics [69]. delayed processing in EDTA tubes increases leukocyte-derived DNA, whereas stabilizing tubes mitigate such drift [70]. These effects extend to genome-wide representation and fragment-length profiles, underscoring the need to report tube type, processing times, and centrifugation schemes. Freeze-thaw cycling, storage temperature, and transport further influence cfDNA stability, with mixed evidence beyond a few cycles, reinforcing the value of aliquoting and prompt processing [69]. Beyond handling, enzymatic exposures and endogenous nuclease activity modulate end-motifs and size spectra, while library-prep and sequencing modality impose systematic capture biases (e.g., dsDNA vs ssDNA protocols; short-read vs long-read), complicating cross-study comparisons [71, 72]. International guidance (e.g., ISO 20186-3) and SPIDIA/European CEN initiatives provide a framework, but consensus, external quality assessment, and method reporting checklists tailored to fragmentomics are urgently needed to improve reproducibility and meta-analytic synthesis.

## The Nucleosome Footprints in cfDNA and Characterizing Methods

Nucleosome positioning patterns, or nucleosome footprints, are also a key focus in cfDNA fragmentomics. There are at least three arguments for this emphasis. First, cfDNA is preferentially cleaved at regions of nucleosomes that are relatively "naked" with histones. This cleavage preference leads to a significant overlap in fragment patterns between cfDNA profiling and nucleosome footprints [73]. Moreover, due to the short half-life of cfDNA (typically 15-30 minutes), cfDNA extracted at any given time represents a snapshot of recent nucleosome dynamics [17]. Second, nucleosomes are known to preferentially occupy transcription start sites (TSS) or transcription factor binding sites (TFBs), regions that are often altered before the onset of gene mutations or abnormal methylation [74, 75]. As such, the study of nucleosome footprints can offer valuable insights into disease progression. At last, while mapping nucleosome positions genome-wide is typically costly and labor-intensive, studying cfDNA provides a more cost-effective approach to understanding nucleosome biology, given that it offers indirect insights into nucleosome positioning without the need for direct genome-wide mapping [76, 77].

The first solid evidence linking nucleosome footprints to disease was presented by Sexton et al., who observed a significant nucleosome redistribution peak 24 hours after the reactivation of Kaposi's sarcoma-associated herpesvirus, with the pattern returning to baseline after 48 hours [78]. This finding underscored the dynamic nature of nucleosome repositioning as part of normal biological processes. A similar pattern was observed in lung and colon adenocarcinoma patients, where Zhu et al. demonstrated that nucleosome redistribution occurred early in cancer initiation but diminished as the disease progressed [79]. These and other studies suggest that transient changes in nucleosome footprints provide a window into specific cellular responses, such as changes in TFs accessibility and chromatin organization [80, 81]. Deeper this study, Maehara et al. identified five principal components derived from 258 cis-regulatory elements that have predictive power for nucleosome positioning and gene expression [82].

Several studies have emphasized the role of DNA methylation in regulating nucleosome positioning. In vertebrate cells, DNA methylation on CpG dinucleotides induces a repressive chromatin state, leading to gene silencing. Consequently, active genes typically exhibit low levels of CpG methylation. This pattern is also reflected in nucleosome footprints, where CpG islands associated with active genes show nucleosome depletion, while inactive regions tend to display increased nucleosome occupancy [83]. For example, Godde et al. examined CGG repeats associated with fragile X syndrome and found that expansion of these repeats disrupted nucleosome assembly on these regions [84]. Further research by Collings et al. provided insights into the mechanisms through which DNA methylation enhances nucleosome affinity to DNA and the histone octamer, specifically in the formation of "M>U nucleosomes" [85]. Additionally, nucleosome footprints are not only shaped by DNA methylation but are also influenced by various post-translational modifications of histones, such as acetylation, phosphorylation, methylation, ubiquitylation, and sumoylation, which can modulate nucleosome positioning and chromatin dynamics in diverse ways [86-91].

Current methods for analyzing nucleosome footprints include MNase-seq, DNASE-seq, and ATAC-seq, which can be used either individually or in combination. All three methods rely on enzymatic cleavage of DNA followed by NGS, with key differences in the enzymes used: MNase, DNASEI, and Tn5 transposase. MNase preferentially cleaves at linker regions, leaving nucleosome-bound DNA fragments intact, while DNASE1 and Tn5 transposase primarily target regions that are not associated with nucleosomes [92]. In essence, MNase-seq is typically used to map nucleosome-occupied DNA, while DNASE-seq and ATAC-seq focus on mapping open chromatin regions. The choice of method largely depends on the research objective. For instance, Teo et al. used MNase-seq to explore the nucleosome landscape of blood cfDNA, noting age-related changes [93]. On the other hand, Zhou et al. employed DNASE-seq to study cfDNA from HCC patients, focusing on regulatory regions enriched in open chromatin [94, 95]. However, these techniques have certain limitations. MNase exhibits a bias against A/T-rich sequences, cleaving them approximately 30 times faster than G/C dinucleotides [96]. Long linker regions are also more prone to MNase cleavage [97, 98]. Similarly, DNASE1 shows a higher affinity for broader minor grooves, creating "DNASE1 hypersensitive sites (DHSs)" at flexible chromatin regions [99, 100]. FAIRE-seq provides an enzyme-free alternative for nucleosome footprinting, utilizing formaldehyde cross-linking to preferentially capture nucleosome-bound DNA, followed by isolation of non-cross-linked DNA for sequencing [101]. FAIRE-seq is less commonly applied to cfDNA studies due to its relatively low signal-to-noise ratio. Additionally, ChIP-seq and the more recent CUT&RUN methods are considered gold standards for identifying transcription factors (TFs) binding to cfDNA in disease contexts [102, 103].

## The Topology Structure in cfDNA and Characterizing Methods

In eukaryotes, DNA exists primarily as a long, double-stranded helix, with the two strands coiled around each other. This DNA undergoes a variety of physical alterations, including stretching, crumpling, and bending, which result in the formation of complex secondary structures known as topological configurations. While these local topological structures do not directly regulate gene expression, their organization facilitates interactions with regulatory molecules [104].

The typical perception of cfDNA topological structures is significantly influenced by DNA sampling and library preparation methods used, which often prioritize the retention of dsDNA. However, with the adoption of ssDNA library preparation methods, the ss-cfDNA fraction has been increasingly detected and is gaining significant attention [42, 43, 105]. Notably, ss-cfDNA is physiologically more susceptible to exonuclease degradation. As a result, the ss-cfDNA fraction tends to increase under pathophysiological conditions, such as cancer. For instance, Wong et al. observed that the ratio of ss-cfDNA to ds-cfDNA was altered in patients with gastric cancer (GC), while it remained stable in healthy individuals. This alteration has been suggested as a potential diagnostic marker for GC, with an AUC of 0.923 [106]. Other utility of ss-cfDNA has also been explored in concepts of colorectal, esophageal, gastric, liver, lung, and ovarian cancers [28, 107]. It is important to note that ss-cfDNA workflow requires an additional heat denaturation step, which converts all DNA molecules to their single-stranded form. Employed solutions include exonuclease degradation, size selection, and specialized adapter ligation strategies [108-110].

Circular DNA is present in most studies of eukaryotes, yet its correlation to the cfDNA was underexplored until recent studies suggested that cancer patients with circular cfDNA exhibit poorer survival outcomes [111]. Key double-strand DNA repair pathways, such as homologous recombination (HR), non-homologous end joining (NHEJ), and R-loop formation, play critical roles in the generation of circular DNA [112]. These mechanisms promote the circularization of chromosomal fragments in response to genomic stress. In the breakage-fusion-bridge (BFB) model, the process is triggered by telomere loss, which leads to end-to-end chromosome fusions and anaphase bridges. These free bridges can break randomly, generating small fragments that can circularize to form circular DNA by repeating this cycle. Alternatively, models such as episome, chromothripsis, and translocation-deletion-amplification formation are also contributory, producing circular DNA as byproducts of faulty repair or replication processes [113-115]. The biological function of circular DNA remains inconclusive, but a common phenomenon observed in yeast is its ability to rapidly and directly increase gene copy number, facilitating adaptation to the changing environment [116]. Similarities can be found in oncogene amplification, MYC family (MYC, MYCN, and MYCL), key drivers for neuroblastomas, is evidenced to be amplified via circular DNA during the early cancer initiation [117, 118]. The circular DNA also accounts for the EGFR, CDK4, and MDM2 amplicon in glioblastoma [119]. Furthermore, the circular DNA also acts as a template for oncogene transcriptions in leukemia, colon cancer and ovarian cancer [119-121].

Circular DNA has also emerged as a promising biomarker, termed circular cfDNA in the context of liquid biopsy. For instance, Fan et al. reported a significantly elevated frequency of circular cfDNA in patients with malignant tumors compared to those with benign tumors [122]. Similarly, Lv et al. analyzed urine samples from patients with advanced chronic kidney disease and identified a disease-associated circular cfDNA profile [123]. However, the closed-loop structure of circular cfDNA poses challenges efficient amplification and reading. To address these issues, innovative methods have been developed. The Circle-seq employs exonuclease digestion prior to library preparation [123-125]. Alternatively, the CIDER-seq utilizes long-read sequencing technologies to directly sequence circular DNA in its entirety without additional amplification [126]. The successful application of these methods has been instrumental in detecting circular cfDNA across various types of cancer, including breast cancer, colorectal cancer, and HCC [127-129].

## cfDNA Fragmentomics in Aging and Related Diseases

Aging-related diseases such as cardiovascular, neurodegenerative, metabolic syndrome, autoimmune diseases, and chronic inflammation are often accompanied by chronic tissue injury and inflammation, and the fragmentation patterns of cfDNA provide unique insights and potential biomarker value in these contexts. In cardiovascular disease, cellular injury releases large amounts of cfDNA into the bloodstream. For example, in patients with acute myocardial infarction (AMI), plasma cfDNA concentrations are significantly elevated, with a standardized mean difference of >3 compared with controls, and the sensitivity and specificity for diagnosing AMI reach 87% and 96%, respectively [130]. Although current research in the cardiovascular field mainly focuses on changes in the overall concentration of cfDNA, fragmentomic analysis is expected to provide more fine-grained information. sWGS sequencing can be used to assess fragment size distribution and tissue-specific methylation characteristics, thereby identifying cardiomyocyte-derived cfDNA fragments, improving diagnostic specificity, and aiding prognostic evaluation [131, 132]. It has been reported that cfDNA concentrations in patients with chronic cardiovascular diseases such as heart failure correlate with disease severity, suggesting that fragmentomic features may be further used for risk stratification [133].

In neurodegenerative diseases, cfDNA fragmentomics is becoming a potential non-invasive diagnostic tool. The plasma cfDNA fragment profiles of Alzheimer’s disease (AD) patients differ significantly from those of healthy elderly controls. Although the total cfDNA concentrations are similar between the two groups, AD patients have significantly lower concentrations of the mononucleosomal peak at ~166 bp and the trinucleosomal peak at ~500 bp (both p<0.05) [134]. This exploratory study demonstrated abnormal cfDNA fragmentation patterns in AD patients. In addition, long-term follow-up studies have shown that high levels of circulating “free fragments” predict early signs of cognitive decline and frailty. In a cohort of cognitively normal elderly individuals (mean age 79), those with higher baseline cfDNA concentrations were more likely to experience cognitive deterioration and frailty within eight years [135]. Researchers speculate that persistently elevated cfDNA fragments may trigger chronic inflammation. This may promote premature aging of tissues and organs, including accelerated aging of the brain. Therefore, cfDNA fragmentomics not only has potential as a diagnostic marker for AD and other diseases but also reflects disease-related neuronal injury and inflammatory states.

Metabolic syndrome and chronic inflammatory states are also associated with abnormal cfDNA fragmentation dynamics. Metabolic disorders such as obesity and type 2 diabetes are often accompanied by persistent low-grade inflammation (“inflammaging”), which may lead to increased apoptosis and elevated cfDNA release. A recent study dynamically monitored cfDNA fragmentomics changes in healthy individuals after meals and found that 30 minutes postprandially, the proportion of short fragments (70-150 bp) in plasma increased significantly while long fragments (151-250 bp) decreased, with the opposite trend observed after two hours [136]. Fragment end sequence analysis revealed decreased DNASE1L3 activity early after feeding, followed later by increased activity of DNASE1 and the apoptotic nuclease DFFB. Meanwhile, metabolic and immune-related gene expressions were upregulated, and the contribution of multiple tissue cell types to cfDNA increased. This evidence suggests that even physiological metabolic stress can significantly affect cfDNA fragment size distributions and nuclease patterns. It indicates that specific cfDNA fragment “fingerprints” may exist in chronic metabolic syndrome to reflect persistent cellular stress and inflammation. Furthermore, in chronic inflammation, cfDNA itself can act as a damage-associated molecular pattern (DAMP) to activate innate immunity; if cfDNA is not promptly cleared, it may sustain positive inflammatory feedback [137, 138]. This provides new avenues for liquid biopsy in metabolic and inflammatory diseases.

Research on cfDNA fragmentomics in autoimmune diseases is more direct. Elevated plasma cfDNA concentrations in systemic lupus erythematosus (SLE) were first reported as early as 1966 [139]. Similar phenomena have been confirmed in rheumatoid arthritis and other diseases, mainly due to excessive cell death (accelerated apoptosis and NETosis) and impaired clearance [140]. Due to technical limitations, earlier studies primarily focused on correlations between cfDNA concentrations and disease activity. However, with advances in genomic and methylomic sequencing, recent studies have begun to explore qualitative changes in cfDNA in autoimmune diseases, such as fragment length distributions, tissue origin, and methylation characteristics [141]. This is similar to the analysis of cfDNA molecular features in cancer liquid biopsy. In SLE and RA patients, accelerated apoptosis releases large amounts of nuclear and mitochondrial DNA, and defective clearance mechanisms allow these fragments to persist in circulation. Neutrophil extracellular traps (NETs) further release intact chromatin into plasma. If not promptly degraded, these can form complexes with antimicrobial proteins. And these complexes may cause capillary occlusion, leading to tissue damage and inflammation. Therefore, cfDNA fragment features (e.g., nucleosomal length patterns, mitochondrial DNA proportion) may serve as strong indicators of disease activity and prognosis in autoimmune disease [142]. Supporting this, mechanistic studies have demonstrated enhanced NETosis in SLE and RA, selectively releasing highly proinflammatory mitochondrial DNA fragments [139]. In summary, in autoimmune and chronic inflammatory environments, cfDNA fragmentomics not only serves as a biomarker but also actively participates in mediating immune dysregulation.

With advancing age, the genome and epigenetic regulation undergo widespread changes, and cfDNA fragmentomics research is revealing some of these regularities [143]. A previous study investigating multiple physiological variables found that age did not significantly affect the basic cfDNA fragment distribution profile [136]. In other words, when preanalytical and sampling conditions were standardized, the average fragment size distribution did not differ significantly among individuals of different ages. However, higher-resolution whole-genome fragmentomic analyses reveal subtle age-related changes. Teo et al. compared cfDNA sequencing from individuals of different age groups (young, elderly, and centenarians) and found that nucleosome footprints derived from cfDNA were remodeled in the elderly [93]. Specifically, the relative abundance of cfDNA decreased at certain genomic regions such as transcription start sites, transcription termination sites, the 5′ ends of L1HS retrotransposons, and dimeric AluY elements, consistent with heterochromatin redistribution during cellular aging. Meanwhile, increasing age and declining health status were also associated with increased diversity in the tissue origins of cfDNA. In elderly individuals, plasma cfDNA contained detectable signals from a wider range of tissues and cell types. This suggests that beyond hematopoietic cells, other tissues increasingly release DNA into the circulation as aging progresses, thereby broadening the tissue footprint of cfDNA. Thus, cfDNA fragmentomics may serve as a non-invasive tool to study age-related epigenetic and tissue homeostasis changes, enabling dynamic monitoring of aging processes.

Immunosenescence also influences cfDNA clearance and fragmentation patterns because macrophage phagocytosis and DNASE activity may decline [143]. Studies show that cfDNA clearance rates are closely related to DNASE1 concentration. In lupus models and patients, exogenous DNASE supplementation accelerates cfDNA clearance, whereas decreased DNase activity delays degradation and leads to enrichment of longer fragments [144]. For example, a genetic variant (R206C) that reduces DNASE1 activity results in a significant increase in the proportion of long cfDNA fragments in plasma. This suggests that in elderly individuals, immunosenescence may prolong cfDNA circulation and alter its composition, resulting in greater persistence of longer, incompletely degraded fragments and chromatin complexes. These uncleared cfDNA fragments may be recognized as foreign by the host, activating innate immunity and sustaining chronic inflammation [135]. “Inflammaging” arises from this persistent low-grade immune activation in the elderly, partly driven by accumulated stimulation from cfDNA. Notably, under physiological conditions cfDNA is not merely a passive byproduct but also plays a role in immune regulation. It had been shown that removing cfDNA from healthy plasma induced monocytes to produce the inflammatory cytokine IL-8, whereas plasma containing normal cfDNA concentrations maintained high expression of immune homeostasis-related genes [144]. Therefore, appropriate cfDNA concentrations and timely clearance are critical for maintaining immune balance. Immunosenescence disrupts this balance, converting cfDNA from a homeostatic signal into a chronic inflammatory stimulus, thereby accelerating tissue aging and disease progression.

Finally, cfDNA fragmentomics also encompasses higher-order chromatin topology. Innovative methods have recently been developed to infer nuclear three-dimensional (3D) chromatin architecture by analyzing cfDNA fragment “co-localization” patterns. For instance, large-scale low-depth sequencing combined with statistical modeling has revealed coordinated fluctuations in cfDNA fragment production across certain genomic regions, suggesting that these regions are spatially proximal within the nucleus [145]. Such cfDNA-based topological analyses provide a window into 3D chromatin architecture in tissues. In oncology, cfDNA fragment co-occurrence patterns have already been explored for detecting abnormal chromatin conformations in early cancer screening. With respect to aging, nuclear chromatin spatial organization undergoes remodeling, and thus these topological cfDNA signals may also reflect age-associated changes. However, studies directly addressing the impact of aging on cfDNA topological signals remain limited, and future research is needed to apply co-localization analysis to aging biology. Overall, cfDNA fragmentomics, as an emerging liquid biopsy field, shows broad application prospects in aging and age-related diseases. It not only aids in non-invasive diagnosis and prognosis but also provides important clues to the molecular landscape of aging [93]. With the advancement of sequencing technologies and bioinformatics analysis, cfDNA fragmentomics is expected to further elucidate the connections between aging and disease, opening new avenues for precision monitoring and intervention in age-related disorders.

## Machine Learning and Deep Learning in cfDNA Fragmentomics Analysis

In recent years, the high-dimensional and inherently noisy nature of cfDNA fragmentomics data has driven widespread adoption of ML/DL approaches to improve diagnostic accuracy and interpretability in liquid biopsy applications. For example, the DELFI framework segments the genome into large bins (~5 Mb) to extract fragmentation features such as the proportion of short fragments, and employs gradient-boosted classifiers to effectively distinguish cancer from non-cancer samples as well as infer tissue of origin [15]. Traditional ML models such as regularized logistic regression and support vector machines (SVMs) have also been successfully applied to binary classification tasks, including early cancer detection and subtype discrimination based on fragment length metrics and coverage variance features. Inferences about nuclease-specific fragmentation patterns have enriched both model accuracy and mechanistic narratives in cfDNA fragmentomics. Yet, without orthogonal evidence such as matched proteomic profiles, enzymatic assays, or functional perturbations, these interpretations risk being overstated [31, 146]. In parallel, low-coverage cfDNA sequencing has enabled extraction of nucleosome footprint signals. Frameworks such as Griffin exploit periodic fragment length patterns around TFBs to reconstruct chromatin accessibility landscapes, facilitating tumor phenotype and regulatory state inference from modest sequencing depths [147].

Beyond conventional ML, DL architectures have begun to eclipse manually engineered features through representation learning. Transformer-based models encode cfDNA fragment end sequences together with genomic context as “fragment sentences” have yielded generalized embeddings across multiple cancer types. The attention weights learned by these models correspond well with known end-motif preferences and cleavage biases, enhancing both prediction performance and biological interpretability [31]. Weakly supervised multiple-instance learning (MIL) frameworks such as DECIDIA further bypass the need for fragment-level labels by leveraging sample-level annotations, demonstrating strong performance in methylation-bisulfite sequencing-based early detection across multicenter cohorts [148]. Moreover, integrative language-model architectures like ACID unify fragmentomic and epigenomic modalities, enabling end-to-end phenotype learning while retaining interpretability via biologically meaningful attention weights (e.g., end motifs, nucleosome footprints) [147]. These advanced methods have also been extended to ultra-low sequencing depths (e.g., ~0.08× coverage), where focused fragment features within protein-coding regions are learned by resource-efficient ML classifiers. While this highlights promising avenues for cost-effective cancer screening, challenges remain related to cohort shifts and sample heterogeneity [149].

Defining appropriate tasks and evaluation strategies is critical for robust deployment. Existing studies span binary and multiclass classification (e.g., healthy vs. cancer, tissue of origin via softmax classifiers) and, in some cases, reconstructed subtype-specific pathways from fragment-derived nucleosome footprints, thereby linking molecular alterations to regulatory phenotypes [150]. In longitudinal settings, fragment-based metrics such as shifts in ultra-short fragment abundance or end-motif patterns indicative of nuclease activity have been coupled with mixed-effects or time-series models to track recurrence and therapy response. Yet, clinical relevance cannot be judged solely by discrimination metrics like AUC; calibration, PPV/NPV at realistic prevalence, and decision-curve analyses are essential to assess real-world benefits. Methodological rigor remains a prerequisite for clinical translation. Best practices include partitioning data into independent training, validation, and external test cohorts; preventing leakage from repeated samples of the same individual; and incorporating multi-center validation to test generalizability [151]. Pre-analytical batch effects from blood collection tubes, preservatives, centrifugation protocols, or library preparation enzymes can significantly distort fragmentomic signatures. Mitigation strategies, such as ComBat-based harmonization or stratified normalization, should be paired with sensitivity analyses to quantify the residual effect of technical variables [29].

Taken together, ML/DL approaches are reshaping cfDNA fragmentomics by enabling models that are sensitive, interpretable, and potentially deployable in clinical workflows. Yet, ensuring their reliability demands more than algorithmic innovation requires robust experimental design, orthogonal biological validation, careful handling of pre-analytical and cohort biases, and transparent evaluation of clinical utility. Representative advances include DELFI [15], Griffin [146], nuclease-mechanism focused end-motif deconvolution [31, 146], Transformer-based end-sequence encoding [148], ACID language-model fusion [147], ultra-low-coverage strategies [149], and each illustrating both the promise and the challenges ahead. Importantly, significant translational gaps remain. Regulatory hurdles require rigorous clinical validation under FDA/EMA frameworks before widespread adoption [152]. The cost-effectiveness and scalability of ML/DL-augmented cfDNA assays also need to be demonstrated, as modeling suggests they are only economically viable if assay costs are substantially reduced [153]. Moreover, true generalizability will require large, prospective multi-center trials to prove robustness across diverse populations and clinical settings. Finally, reproducibility challenges persist, as cohort shifts, batch effects, and heterogeneity can undermine model performance [154]. Addressing these pitfalls will necessitate concerted multidisciplinary efforts, combining computational rigor with standardized workflows and collaborative validation, to ensure that the promise of ML/DL in cfDNA fragmentomics can ultimately be realized in patient care.

## Discussion

cfDNA fragmentomics explores the unique physical properties of cfDNA fragments, including size, shape, and integrity, providing a novel and nuanced approach for detecting genetic variants. This is particularly valuable for early-stage diseases that may not exhibit significant genome-wide alterations. This review summarized the key characteristics of cfDNA fragmentomics, its biogenesis, biological and pathological functions, as well as the detection strategies. We also discussed the variations among characteristic subtypes and their impact on analytical approaches. Collectively, this review highlighted recent advances in publications and applications, offering readers a comprehensive understanding of the topic. Despite these developments, several key questions remain unresolved.

First, unlike non-cfDNA fragmentomics analysis that typically detects variants from plasma, cfDNA fragmentomics has a primary focus on urine samples. From a biological perspective, this shift is attributed to the higher nuclease activity and concentration in urine which accelerates DNA fragmentation, including that of cfDNA [155]. Besides, elevated sodium and other electrolytes create a high-salt environment in urine, it disrupts histone-DNA coiling and further destabilizes the chromatin structure [156]. These factors make cfDNA fragmentomics promising diagnostic targets for diseases that shed DNA molecules into the urinary tract, such as bladder cancer, prostate cancer, and renal cell carcinoma. However, this raises crucial questions about the effects of the local environment, or niche, on cfDNA fragmentomics patterns. Are the patterns observed in urine comparable to those in other liquid biopsy sources, such as saliva or cerebrospinal fluid? Furthermore, do the cfDNA fragmentomics patterns observed in vitro align with those found in vivo, or do significant differences exist?

Second, while this review and most current cfDNA fragmentomics focuses on primary DNA structure, higher-order structures such as G-quadruplexes (G4), Z-DNA, cruciform, and triplex DNA have received comparatively less attention [157]. These structures form complex conformations that protect cfDNA from enzymatic degradation and preserve their structural integrity and informational content in liquid biopsies. More importantly, they are involved in key biological processes including transcription, replication, and repair, with alterations in their dynamics linked to disease onset and progression. Analyzing these higher-order structures could offer significant advantages over basic cfDNA fragmentomics because they may provide deeper insights into genomic stability and cellular processes. Nevertheless, capturing and studying these structures remains challenging. Their transient nature and dependence on specific physiological conditions make it difficult to stabilize in vitro, and current detection technologies, such as sequencing and PCR, are optimized for linear DNA and struggle with such complex conformations. Developing more sensitive, high-resolution tools to reliably identify and study these structures will be crucial for future research.

Third, cfDNA fragmentomics and non-fragmentomics analyses are not mutually exclusive but can complement each other, filling in gaps that a single approach might overlook. On one hand, cfDNA fragmentomics examines the physical properties of cfDNA, yet it does not provide information about genetic or epigenetic alterations that may drive disease. On the other hand, cfDNA non-fragmentomics focuses on sequence-specific information but cannot capture the structural and fragmentation patterns that reflect broader genomic instability or cellular processes. Integrating cfDNA fragmentomics and non-fragmentomics will be an innovative direction for developing more precise diagnostic tools. One potential application perhaps being to combine traditional NGS methods for mutation detection with additional approaches to identify tissue origin based on fragmentomics patterns [158]. A key challenge in this integration is data fusion in multi-modal models, where aligning high-dimensional fragmentomics with non-fragmentomics features requires advanced computational frameworks to prevent information loss or overfitting. Current successful integration has still been limited to simple features, such as combining cfDNA copy number with fragment size profiles [158]. Looking forward, optimized analytical frameworks capable of simultaneously assessing both fragmentomics and sequence-specific properties may set new standards for cfDNA-based diagnostics.

Importantly, the growing integration of ML/DL into cfDNA fragmentomics pipelines represents a transformative direction for the field. Feature-based ML frameworks, such as DELFI, and advanced representation learning approaches, including transformer-based end-motif models and multiple-instance learning architectures, have demonstrated the potential to extract highly discriminative and biologically interpretable signals from inherently noisy fragmentomic data. Moreover, ultra-low coverage strategies combined with cost-sensitive learning, robust batch effect correction, and biologically informed feature validation are expanding the feasibility of fragmentomics for large-scale, cost-effective cancer screening. However, low-coverage sequencing, such as sWGS, LP-WGS, and LC-WGS, risks overfitting due to sparse data and noise, potentially leading to false positives in cfDNA fragmentomics profiling. To mitigate this, strategies like cross-validation, regularization (e.g., L1/L2 penalties), and ensemble methods, as often employed in the DELFI framework [159]. Embedding such computational frameworks into multi-modal analyses that unite fragmentomic and non-fragmentomic features could markedly enhance sensitivity, specificity, and translational applicability in diverse clinical contexts.

In conclusion, cfDNA fragmentomics has the potential to reshape the field of liquid biopsy diagnostics, offering fresh perspectives on disease detection and monitoring. While substantial progress has been made, the field presents both challenges and opportunities. Collaborative efforts to refine and expand methodologies, along with the development of novel, high-resolution technologies, will be essential to fully harness the diagnostic potential of cfDNA fragmentomics. As our understanding deepens, the convergence of advanced computational modeling with biological insight will be critical for enhancing the precision and scope of diagnostic tools, ultimately facilitating earlier and more accurate disease detection and advancing the field of personalized medicine.

## References

[1] Fan HC, Blumenfeld YJ, Chitkara U, Hudgins L, Quake SR (2008). Noninvasive diagnosis of fetal aneuploidy by shotgun sequencing DNA from maternal blood. Proc Natl Acad Sci USA, 105:16266-16271. 10.1073/pnas.0808319105 PMC2562413 18838674

[2] Scotchman E, Chandler NJ, Mellis R, Chitty LS (2020). Noninvasive Prenatal Diagnosis of Single-Gene Diseases: The Next Frontier. Clin Chem, 66:53-60. 10.1373/clinchem.2019.304238 31843868

[3] Zviran A, Schulman RC, Shah M, Hill STK, Deochand S, Khamnei CC, et al. (2020). Genome-wide cell-free DNA mutational integration enables ultra-sensitive cancer monitoring. Nat Med, 26:1114-1124. 10.1038/s41591-020-0915-3 PMC8108131 32483360

[4] Phallen J, Sausen M, Adleff V, Leal A, Hruban C, White J, et al. (2017). Direct detection of early-stage cancers using circulating tumor DNA. Sci Transl Med, 9:eaan2415. 10.1126/scitranslmed.aan2415 PMC6714979 28814544

[5] Moss J, Zick A, Grinshpun A, Carmon E, Maoz M, Ochana BL, et al. (2020). Circulating breast-derived DNA allows universal detection and monitoring of localized breast cancer. Ann Oncol, 31:395-403. 10.1016/j.annonc.2019.11.014 32067681

[6] Wan JC, Massie C, Garcia-Corbacho J, Mouliere F, Brenton JD, Caldas C, et al. (2017). Liquid biopsies come of age: towards implementation of circulating tumour DNA. Nat Rev Cancer, 17:223-238. 10.1038/nrc.2017.7 28233803

[7] Devonshire AS, Whale AS, Gutteridge A, Jones G, Cowen S, Foy CA, et al. (2014). Towards standardisation of cell-free DNA measurement in plasma: controls for extraction efficiency, fragment size bias and quantification. Anal Bioanal Chem, 406:6499-6512. 10.1007/s00216-014-7835-3 PMC4182654 24853859

[8] Heitzer E, Ulz P, Geigl JB (2015). Circulating tumor DNA as a liquid biopsy for cancer. Clin Chem, 61:112-123. 10.1373/clinchem.2014.222679 25388429

[9] Chan HT, Chin YM, Nakamura Y, Low S-K (2020). Clonal Hematopoiesis in Liquid Biopsy: From Biological Noise to Valuable Clinical Implications. Cancers, 12:2277. 10.3390/cancers12082277 PMC7463455 32823942

[10] Zink F, Stacey SN, Norddahl GL, Frigge ML, Magnusson OT, Jonsdottir I, et al. (2017). Clonal hematopoiesis, with and without candidate driver mutations, is common in the elderly. Blood, 130:742-752. 10.1182/blood-2017-02-769869 PMC5553576 28483762

[11] McKerrell T, Park N, Moreno T, Grove CS, Ponstingl H, Stephens J, et al. (2015). Leukemia-associated somatic mutations drive distinct patterns of age-related clonal hemopoiesis. Cell Rep, 10:1239-1245. 10.1016/j.celrep.2015.02.005 PMC4542313 25732814

[12] Weaver VM, Gilbert P (2004). Watch thy neighbor: cancer is a communal affair. J Cell Sci, 117:1287-1290. 10.1242/jcs.01137 15020668

[13] Razavi P, Li BT, Brown DN, Jung B, Hubbell E, Shen R, et al. (2019). High-intensity sequencing reveals the sources of plasma circulating cell-free DNA variants. Nat Med, 25:1928-1937. 10.1038/s41591-019-0652-7 PMC7061455 31768066

[14] Ivanov M, Baranova A, Butler T, Spellman P, Mileyko V (2015). Non-random fragmentation patterns in circulating cell-free DNA reflect epigenetic regulation. BMC Genomics, 16 Suppl 13:S1. 10.1186/1471-2164-16-S13-S1 PMC4686799 26693644

[15] Cristiano S, Leal A, Phallen J, Fiksel J, Adleff V, Bruhm DC, et al. (2019). Genome-wide cell-free DNA fragmentation in patients with cancer. Nature, 570:385-389. 10.1038/s41586-019-1272-6 PMC6774252 31142840

[16] Ying H, Fengying S, Feng H, Yanhong W, Xianru X, Xiaolei T (2021). Diagnostic value of quantification of circulating free DNA for gall bladder cancer using a chemiluminescence DNA biosensor system based on DNA G-quadruplex/ hemin enzyme. Transl Oncol, 14:100928. 10.1016/j.tranon.2020.100928 PMC7679247 33212417

[17] Volik S, Alcaide M, Morin RD, Collins C (2016). Cell-free DNA (cfDNA): Clinical Significance and Utility in Cancer Shaped By Emerging Technologies. Mol Cancer Res, 14:898-908. 10.1158/1541-7786.MCR-16-0044 27422709

[18] Board RE, Williams VS, Knight L, Shaw J, Greystoke A, Ranson M, et al. (2008). Isolation and extraction of circulating tumor DNA from patients with small cell lung cancer. Ann N Y Acad Sci, 1137:98-107. 10.1196/annals.1448.020 18837931

[19] Li Y, Zimmermann B, Rusterholz C, Kang A, Holzgreve W, Hahn S (2004). Size separation of circulatory DNA in maternal plasma permits ready detection of fetal DNA polymorphisms. Clin Chem, 50:1002-1011. 10.1373/clinchem.2003.029835 15073090

[20] Jiang P, Lo YMD (2016). The Long and Short of Circulating Cell-Free DNA and the Ins and Outs of Molecular Diagnostics. Trends Genet, 32:360-371. 10.1016/j.tig.2016.03.009 27129983

[21] Jiang P, Chan CW, Chan KC, Cheng SH, Wong J, Wong VW, et al. (2015). Lengthening and shortening of plasma DNA in hepatocellular carcinoma patients. Proc Natl Acad Sci USA, 112:E1317-1325. 10.1073/pnas.1500076112 PMC4372002 25646427

[22] Yu SCY, Jiang P, Peng W, Cheng SH, Cheung YTT, Tse OYO, et al. (2021). Single-molecule sequencing reveals a large population of long cell-free DNA molecules in maternal plasma. Proc Natl Acad Sci USA, 118:e2114937118. 10.1073/pnas.2114937118 PMC8685924 34873045

[23] Zheng YW, Chan KC, Sun H, Jiang P, Su X, Chen EZ, et al. (2012). Nonhematopoietically derived DNA is shorter than hematopoietically derived DNA in plasma: a transplantation model. Clin Chem, 58:549-558. 10.1373/clinchem.2011.169318 22052939

[24] Lo YM, Chan KC, Sun H, Chen EZ, Jiang P, Lun FM, et al. (2010). Maternal plasma DNA sequencing reveals the genome-wide genetic and mutational profile of the fetus. Sci Transl Med, 2:61ra91. 10.1126/scitranslmed.3001720 21148127

[25] Lun FM, Chiu RW, Sun K, Leung TY, Jiang P, Chan KC, et al. (2013). Noninvasive prenatal methylomic analysis by genomewide bisulfite sequencing of maternal plasma DNA. Clin Chem, 59:1583-1594. 10.1373/clinchem.2013.212274 23857673

[26] Jensen TJ, Kim SK, Zhu Z, Chin C, Gebhard C, Lu T, et al. (2015). Whole genome bisulfite sequencing of cell-free DNA and its cellular contributors uncovers placenta hypomethylated domains. Genome Biol, 16:78. 10.1186/s13059-015-0645-x PMC4427941 25886572

[27] Yu SC, Chan KC, Zheng YW, Jiang P, Liao GJ, Sun H, et al. (2014). Size-based molecular diagnostics using plasma DNA for noninvasive prenatal testing. Proc Natl Acad Sci USA, 111:8583-8588. 10.1073/pnas.1406103111 PMC4060699 24843150

[28] Wang F, Li X, Li M, Liu W, Lu L, Li Y, et al. (2024). Ultra-short cell-free DNA fragments enhance cancer early detection in a multi-analyte blood test combining mutation, protein and fragmentomics. Clin Chem Lab Med, 62:168-177. 10.1515/cclm-2023-0541 37678194

[29] Park SH, Lee HJ, Kim TI, Lee J, Han SY, Seo HI, et al. (2024). Ultrashort Cell-Free DNA Fragments and Vimentin-Positive Circulating Tumor Cells for Predicting Early Recurrence in Patients with Biliary Tract Cancer. Diagnostics, 14(21):2462. 10.3390/diagnostics14212462 PMC11544859 39518429

[30] Salas S, Phuong LN, Garcia J-C, Greillier L, Gaudy-Marqueste C, Deville J-L, et al. (2024). Long cell-free DNA fragments predict early-progression in patients with advanced or metastatic cancer treated with immune-checkpoint inhibition. J Clin Oncol, 42:e14550.

[31] Shen H, Yang M, Liu J, Chen K, Li X (2024). Development of a deep learning model for cancer diagnosis by inspecting cell-free DNA end-motifs. NPJ Precis Oncol, 8:160. 10.1038/s41698-024-00635-5 PMC11283569 39068267

[32] Sun K, Jiang P, Wong AIC, Cheng YKY, Cheng SH, Zhang H, et al. (2018). Size-tagged preferred ends in maternal plasma DNA shed light on the production mechanism and show utility in noninvasive prenatal testing. Proc Natl Acad Sci USA, 115:E5106-e5114. 10.1073/pnas.1804134115 PMC5984542 29760053

[33] Ding SC, Chan RWY, Peng W, Huang L, Zhou Z, Hu X, et al. (2022). Jagged Ends on Multinucleosomal Cell-Free DNA Serve as a Biomarker for Nuclease Activity and Systemic Lupus Erythematosus. Clin Chem, 68:917-926. 10.1093/clinchem/hvac050 35587043

[34] Cheng J, Swarup N, Li F, Kordi M, Lin C-C, Yang S-C, et al. (2023). Distinct Features of Plasma Ultrashort Single-Stranded Cell-Free DNA as Biomarkers for Lung Cancer Detection. Clin Chem, 69:1270-1282. 10.1093/clinchem/hvad131 PMC10644908 37725931

[35] Fiorini E, Malinova A, Schreyer D, Pasini D, Bevere M, Alessio G, et al. (2025). MYC ecDNA promotes intratumour heterogeneity and plasticity in PDAC. Nature, 640(8059):811-820. 10.1038/s41586-025-08721-9 PMC12003172 40074906

[36] Sasaki M, Kobayashi T (2025). Transcription near arrested DNA replication forks triggers ribosomal DNA copy number changes. Nucleic Acids Research, 53(3):gkaf014. 10.1093/nar/gkaf014 PMC11760980 39876709

[37] Kumar P, Dillon LW, Shibata Y, Jazaeri AA, Jones DR, Dutta A (2017). Normal and Cancerous Tissues Release Extrachromosomal Circular DNA (eccDNA) into the Circulation. Mol Cancer Res, 15:1197-1205. 10.1158/1541-7786.MCR-17-0095 PMC5581709 28550083

[38] Jacob DR, Guiblet WM, Mamayusupova H, Shtumpf M, Ciuta I, Ruje L, et al. (2024). Nucleosome reorganisation in breast cancer tissues. Clinical Epigenetics, 16:50. 10.1186/s13148-024-01656-4 PMC10986098 38561804

[39] De Sarkar N, Patton RD, Doebley AL, Hanratty B, Adil M, Kreitzman AJ, et al. (2023). Nucleosome Patterns in Circulating Tumor DNA Reveal Transcriptional Regulation of Advanced Prostate Cancer Phenotypes. Cancer Discov, 13:632-653. 10.1158/2159-8290.CD-22-0692 PMC9976992 36399432

[40] Chan KC, Jiang P, Sun K, Cheng YK, Tong YK, Cheng SH, et al. (2016). Second generation noninvasive fetal genome analysis reveals de novo mutations, single-base parental inheritance, and preferred DNA ends. Proc Natl Acad Sci USA, 113:E8159-e8168. 10.1073/pnas.1615800113 PMC5167168 27799561

[41] Mouliere F, Chandrananda D, Piskorz AM, Moore EK, Morris J, Ahlborn LB, et al. (2018). Enhanced detection of circulating tumor DNA by fragment size analysis. Sci Transl Med, 10:466. 10.1126/scitranslmed.aat4921 PMC6483061 30404863

[42] Burnham P, Kim MS, Agbor-Enoh S, Luikart H, Valantine HA, Khush KK, et al. (2016). Single-stranded DNA library preparation uncovers the origin and diversity of ultrashort cell-free DNA in plasma. Sci Rep, 6:27859. 10.1038/srep27859 PMC4906518 27297799

[43] Snyder MW, Kircher M, Hill AJ, Daza RM, Shendure J (2016). Cell-free DNA Comprises an In Vivo Nucleosome Footprint that Informs Its Tissues-Of-Origin. Cell, 164:57-68. 10.1016/j.cell.2015.11.050 PMC4715266 26771485

[44] Hudecova I, Smith CG, Hänsel-Hertsch R, Chilamakuri CS, Morris JA, Vijayaraghavan A, et al. (2022). Characteristics, origin, and potential for cancer diagnostics of ultrashort plasma cell-free DNA. Genome Res 32:215-227. 10.1101/gr.275691.121 PMC8805718 34930798

[45] Liu X, Liu L, Ji Y, Li C, Wei T, Yang X, et al. (2019). Enrichment of short mutant cell-free DNA fragments enhanced detection of pancreatic cancer. eBioMedicine, 41:345-356. 10.1016/j.ebiom.2019.02.010 PMC6442234 30857943

[46] Zhu J, Huang J, Zhang P, Li Q, Kohli M, Huang C-C, et al. (2020). Advantages of single-stranded DNA over double-stranded DNA library preparation for capturing cell-free tumor DNA in plasma. Mol Diagn Ther, 24:95-101. 10.1007/s40291-019-00429-7 PMC7216418 31654347

[47] Moser T, Ulz P, Zhou Q, Perakis S, Geigl JB, Speicher MR, et al. (2017). Single-Stranded DNA Library Preparation Does Not Preferentially Enrich Circulating Tumor DNA. Clin Chem, 63:1656-1659. 10.1373/clinchem.2017.277988 28807979

[48] Smith CG, Moser T, Mouliere F, Field-Rayner J, Eldridge M, Riediger AL, et al. (2020). Comprehensive characterization of cell-free tumor DNA in plasma and urine of patients with renal tumors. Genome Med, 12:23. 10.1186/s13073-020-00723-8 PMC7048087 32111235

[49] Chan KCA, Jiang P, Sun K, Cheng YKY, Tong YK, Cheng SH, et al. (2016). Second generation noninvasive fetal genome analysis reveals de novo mutations, single-base parental inheritance, and preferred DNA ends. Proc Natl Acad Sci USA, 113:E8159-E8168. 10.1073/pnas.1615800113 PMC5167168 27799561

[50] Han DSC, Ni M, Chan RWY, Chan VWH, Lui KO, Chiu RWK, et al. (2020). The Biology of Cell-free DNA Fragmentation and the Roles of DNASE1, DNASE1L3, and DFFB. Am J Hum Genet, 106:202-214. 10.1016/j.ajhg.2020.01.008 PMC7010979 32004449

[51] Jiang P, Sun K, Tong YK, Cheng SH, Cheng THT, Heung MMS, et al. (2018). Preferred end coordinates and somatic variants as signatures of circulating tumor DNA associated with hepatocellular carcinoma. Proc Natl Acad Sci USA, 115:E10925-e10933. 10.1073/pnas.1814616115 PMC6243268 30373822

[52] Chandrananda D, Thorne NP, Bahlo M (2015). High-resolution characterization of sequence signatures due to non-random cleavage of cell-free DNA. BMC Med Genomics, 8:29. 10.1186/s12920-015-0107-z PMC4469119 26081108

[53] Serpas L, Chan RWY, Jiang P, Ni M, Sun K, Rashidfarrokhi A, et al. (2019). Dnase1l3 deletion causes aberrations in length and end-motif frequencies in plasma DNA. Proc Natl Acad Sci USA, 116:641-649. 10.1073/pnas.1815031116 PMC6329986 30593563

[54] Jiang P, Sun K, Peng W, Cheng SH, Ni M, Yeung PC, et al. (2020). Plasma DNA End-Motif Profiling as a Fragmentomic Marker in Cancer, Pregnancy, and Transplantation. Cancer Discov, 10:664-673. 10.1158/2159-8290.CD-19-0622 32111602

[55] Li W, Nakano H, Fan W, Li Y, Sil P, Nakano K, et al. (2023). DNASE1L3 enhances antitumor immunity and suppresses tumor progression in colon cancer. JCI Insight, 8:17. 10.1172/jci.insight.168161 PMC10544201 37581941

[56] Liu J, Yi J, Zhang Z, Cao D, Li L, Yao Y (2021). Deoxyribonuclease 1-like 3 may be a potential prognostic biomarker associated with immune infiltration in colon cancer. Aging (Albany NY), 13:16513-16526. 10.18632/aging.203173 PMC8266351 34157681

[57] Napirei M, Ludwig S, Mezrhab J, Klöckl T, Mannherz HG (2009). Murine serum nucleases--contrasting effects of plasmin and heparin on the activities of DNase1 and DNase1-like 3 (DNase1l3). Febs j, 276:1059-1073. 10.1111/j.1742-4658.2008.06849.x 19154352

[58] Jiang P, Xie T, Ding SC, Zhou Z, Cheng SH, Chan RWY, et al. (2020). Detection and characterization of jagged ends of double-stranded DNA in plasma. Genome Res, 30:1144-1153. 10.1101/gr.261396.120 PMC7462074 32801148

[59] Burnham P, Dadhania D, Heyang M, Chen F, Westblade LF, Suthanthiran M, et al. (2018). Urinary cell-free DNA is a versatile analyte for monitoring infections of the urinary tract. Nat Commun, 9:2412. 10.1038/s41467-018-04745-0 PMC6010457 29925834

[60] Chen M, Chan RWY, Cheung PPH, Ni M, Wong DKL, Zhou Z, et al. (2022). Fragmentomics of urinary cell-free DNA in nuclease knockout mouse models. PLoS Genet, 18:e1010262. 10.1371/journal.pgen.1010262 PMC9258866 35793278

[61] Zhou Z, Cheng SH, Ding SC, Heung MMS, Xie T, Cheng THT, et al. (2021). Jagged Ends of Urinary Cell-Free DNA: Characterization and Feasibility Assessment in Bladder Cancer Detection. Clin Chem, 67:621-630. 10.1093/clinchem/hvaa325 33604652

[62] Kleiman L, Huang RC (1972). Reconstitution of chromatin. The sequential binding of histones to DNA in the presence of salt and urea. J Mol Biol, 64:1-8. 10.1016/0022-2836(72)90317-8 5062731

[63] Markus H, Zhao J, Contente-Cuomo T, Stephens MD, Raupach E, Odenheimer-Bergman A, et al. (2021). Analysis of recurrently protected genomic regions in cell-free DNA found in urine. Sci Transl Med, 13:eaaz3088. 10.1126/scitranslmed.aaz3088 PMC9258975 33597261

[64] Eulitz D, Mannherz HG (2007). Inhibition of deoxyribonuclease I by actin is to protect cells from premature cell death. Apoptosis, 12:1511-1521. 10.1007/s10495-007-0078-4 17468836

[65] Kishi K, Yasuda T, Ikehara Y, Sawazaki K, Sato W, Iida R (1990). Human serum deoxyribonuclease I (DNase I) polymorphism: pattern similarities among isozymes from serum, urine, kidney, liver, and pancreas. Am J Hum Genet, 47:121-126. PMC1683738 2349940

[66] Crosetto N, Mitra A, Silva MJ, Bienko M, Dojer N, Wang Q, et al. (2013). Nucleotide-resolution DNA double-strand break mapping by next-generation sequencing. Nat Methods, 10:361-365. 10.1038/nmeth.2408 PMC3651036 23503052

[67] Canela A, Sridharan S, Sciascia N, Tubbs A, Meltzer P, Sleckman BP, et al. (2016). DNA Breaks and End Resection Measured Genome-wide by End Sequencing. Mol Cell, 63:898-911. 10.1016/j.molcel.2016.06.034 PMC6299834 27477910

[68] Napirei M, Wulf S, Eulitz D, Mannherz HG, Kloeckl T (2005). Comparative characterization of rat deoxyribonuclease 1 (Dnase1) and murine deoxyribonuclease 1-like 3 (Dnase1l3). Biochem J, 389:355-364. 10.1042/BJ20042124 PMC1175112 15796714

[69] Batool SM, Hsia T, Beecroft A, Lewis B, Ekanayake E, Rosenfeld Y, et al. (2023). Extrinsic and intrinsic preanalytical variables affecting liquid biopsy in cancer. Cell Rep Med, 4:101196. 10.1016/j.xcrm.2023.101196 PMC10591035 37725979

[70] Gianni C, Palleschi M, Merloni F, Di Menna G, Sirico M, Sarti S, et al. (2022). Cell-Free DNA Fragmentomics: A Promising Biomarker for Diagnosis, Prognosis and Prediction of Response in Breast Cancer. Int J Mol Sci, 23:22. 10.3390/ijms232214197 PMC9695769 36430675

[71] Greytak SR, Engel KB, Parpart-Li S, Murtaza M, Bronkhorst AJ, Pertile MD, et al. (2020). Harmonizing Cell-Free DNA Collection and Processing Practices through Evidence-Based Guidance. Clin Cancer Res, 26:3104-3109. 10.1158/1078-0432.CCR-19-3015 PMC7334102 32122922

[72] Peng H, Pan M, Zhou Z, Chen C, Xing X, Cheng S, et al. (2024). The impact of preanalytical variables on the analysis of cell-free DNA from blood and urine samples. Front Cell Dev Biol, 12:1385041. 10.3389/fcell.2024.1385041 PMC11111958 38784382

[73] Heitzer E, Auinger L, Speicher MR (2020). Cell-Free DNA and Apoptosis: How Dead Cells Inform About the Living. Trends Mol Med, 26:519-528. 10.1016/j.molmed.2020.01.012 32359482

[74] Pich O, Muiños F, Sabarinathan R, Reyes-Salazar I, Gonzalez-Perez A, Lopez-Bigas N (2018). Somatic and Germline Mutation Periodicity Follow the Orientation of the DNA Minor Groove around Nucleosomes. Cell, 175:1074-1087.e1018. 10.1016/j.cell.2018.10.004 30388444

[75] Li C, Luscombe NM (2020). Nucleosome positioning stability is a modulator of germline mutation rate variation across the human genome. Nat Commun, 11:1363. 10.1038/s41467-020-15185-0 PMC7070026 32170069

[76] Schones DE, Cui K, Cuddapah S, Roh TY, Barski A, Wang Z, et al. (2008). Dynamic regulation of nucleosome positioning in the human genome. Cell, 132:887-898. 10.1016/j.cell.2008.02.022 PMC10894452 18329373

[77] Teif VB, Vainshtein Y, Caudron-Herger M, Mallm JP, Marth C, Höfer T, et al. (2012). Genome-wide nucleosome positioning during embryonic stem cell development. Nat Struct Mol Biol, 19:1185-1192. 10.1038/nsmb.2419 23085715

[78] Sexton BS, Avey D, Druliner BR, Fincher JA, Vera DL, Grau DJ, et al. (2014). The spring-loaded genome: nucleosome redistributions are widespread, transient, and DNA-directed. Genome Res, 24:251-259. 10.1101/gr.160150.113 PMC3912415 24310001

[79] Druliner BR, Vera D, Johnson R, Ruan X, Apone LM, Dimalanta ET, et al. (2016). Comprehensive nucleosome mapping of the human genome in cancer progression. Oncotarget, 7:13429-13445. 10.18632/oncotarget.6811 PMC4924652 26735342

[80] Wolffe AP, Kurumizaka H (1998). The nucleosome: a powerful regulator of transcription. Prog Nucleic Acid Res Mol Biol, 61:379-422. 10.1016/s0079-6603(08)60832-6 9752726

[81] Zhu F, Farnung L, Kaasinen E, Sahu B, Yin Y, Wei B, et al. (2018). The interaction landscape between transcription factors and the nucleosome. Nature, 562:76-81. 10.1038/s41586-018-0549-5 PMC6173309 30250250

[82] Maehara K, Ohkawa Y (2016). Exploration of nucleosome positioning patterns in transcription factor function. Sci Rep, 6:19620. 10.1038/srep19620 PMC4726364 26790608

[83] Deaton AM, Bird A (2011). CpG islands and the regulation of transcription. Genes Dev, 25:1010-1022. 10.1101/gad.2037511 PMC3093116 21576262

[84] Godde JS, Kass SU, Hirst MC, Wolffe AP (1996). Nucleosome assembly on methylated CGG triplet repeats in the fragile X mental retardation gene 1 promoter. J Biol Chem, 271:24325-24328. 10.1074/jbc.271.40.24325 8798682

[85] Collings CK, Waddell PJ, Anderson JN (2013). Effects of DNA methylation on nucleosome stability. Nucleic Acids Res, 41:2918-2931. 10.1093/nar/gks893 PMC3597673 23355616

[86] Eberharter A, Becker PB (2002). Histone acetylation: a switch between repressive and permissive chromatin. Second in review series on chromatin dynamics. EMBO Rep, 3:224-229. 10.1093/embo-reports/kvf053 PMC1084017 11882541

[87] Banerjee T, Chakravarti D (2011). A peek into the complex realm of histone phosphorylation. Mol Cell Biol, 31:4858-4873. 10.1128/MCB.05631-11 PMC3233023 22006017

[88] Miller JL, Grant PA (2013). The role of DNA methylation and histone modifications in transcriptional regulation in humans. Subcell Biochem, 61:289-317. 10.1007/978-94-007-4525-4_13 PMC6611551 23150256

[89] Luo A, Kong J, Chen J, Xiao X, Lan J, Li X, et al. (2023). H2B ubiquitination recruits FACT to maintain a stable altered nucleosome state for transcriptional activation. Nat Commun, 14:741. 10.1038/s41467-023-36467-3 PMC9918737 36765085

[90] Ryu HY, Hochstrasser M (2021). Histone sumoylation and chromatin dynamics. Nucleic Acids Res, 49:6043-6052. 10.1093/nar/gkab280 PMC8216275 33885816

[91] Kim TH, Nosella ML, Bolik-Coulon N, Harkness RW, Huang SK, Kay LE (2023). Correlating histone acetylation with nucleosome core particle dynamics and function. Proc Natl Acad Sci USA, 120:e2301063120. 10.1073/pnas.2301063120 PMC10104578 37011222

[92] Klein DC, Hainer SJ (2020). Genomic methods in profiling DNA accessibility and factor localization. Chromosome Res, 28:69-85. 10.1007/s10577-019-09619-9 PMC7125251 31776829

[93] Teo YV, Capri M, Morsiani C, Pizza G, Faria AMC, Franceschi C, et al. (2019). Cell-free DNA as a biomarker of aging. Aging Cell, 18:e12890. 10.1111/acel.12890 PMC6351822 30575273

[94] Zhou X, Zheng H, Fu H, Dillehay McKillip KL, Pinney SM, Liu Y (2022). CRAG: de novo characterization of cell-free DNA fragmentation hotspots in plasma whole-genome sequencing. Genome Med, 14:138. 10.1186/s13073-022-01141-8 PMC9733064 36482487

[95] Lambert SA, Jolma A, Campitelli LF, Das PK, Yin Y, Albu M, et al. (2018). The Human Transcription Factors. Cell, 172:650-665. 10.1016/j.cell.2018.01.029 PMC12908702 29425488

[96] Dingwall C, Lomonossoff GP, Laskey RA (1981). High sequence specificity of micrococcal nuclease. Nucleic Acids Res, 9:2659-2673. 10.1093/nar/9.12.2659 PMC326883 6269057

[97] Shen CH, Allan J (2021). MNase Digestion Protection Patterns of the Linker DNA in Chromatosomes. Cells, 10(9):2239. 10.3390/cells10092239 PMC8469290 34571888

[98] Rizzo JM, Bard JE, Buck MJ (2012). Standardized collection of MNase-seq experiments enables unbiased dataset comparisons. BMC Mol Biol, 13:15. 10.1186/1471-2199-13-15 PMC3464627 22559821

[99] Jin W, Tang Q, Wan M, Cui K, Zhang Y, Ren G, et al. (2015). Genome-wide detection of DNase I hypersensitive sites in single cells and FFPE tissue samples. Nature, 528:142-146. 10.1038/nature15740 PMC4697938 26605532

[100] Meuleman W, Muratov A, Rynes E, Halow J, Lee K, Bates D, et al. (2020). Index and biological spectrum of human DNase I hypersensitive sites. Nature, 584:244-251. 10.1038/s41586-020-2559-3 PMC7422677 32728217

[101] Giresi PG, Kim J, McDaniell RM, Iyer VR, Lieb JD (2007). FAIRE (Formaldehyde-Assisted Isolation of Regulatory Elements) isolates active regulatory elements from human chromatin. Genome Res, 17:877-885. 10.1101/gr.5533506 PMC1891346 17179217

[102] Rao S, Han AL, Zukowski A, Kopin E, Sartorius CA, Kabos P, et al. (2022). Transcription factor-nucleosome dynamics from plasma cfDNA identifies ER-driven states in breast cancer. Sci Adv, 8:eabm4358. 10.1126/sciadv.abm4358 PMC9401618 36001652

[103] Trier Maansson C, Meldgaard P, Stougaard M, Nielsen AL, Sorensen BS (2023). Cell-free chromatin immunoprecipitation can determine tumor gene expression in lung cancer patients. Mol Oncol, 17:722-736. 10.1002/1878-0261.13394 PMC10158780 36825535

[104] Chen LF, Long HK (2023). Topology regulatory elements: From shaping genome architecture to gene regulation. Curr Opin Struct Biol, 83:102723. 10.1016/j.sbi.2023.102723 PMC7615376 37931379

[105] Wu DC, Lambowitz AM (2017). Facile single-stranded DNA sequencing of human plasma DNA via thermostable group II intron reverse transcriptase template switching. Sci Rep, 7:8421. 10.1038/s41598-017-09064-w PMC5566474 28827600

[106] Huang X, Zhao Q, An X, Pan J, Zhao L, Shen L, et al. (2020). The Ratio of ssDNA to dsDNA in Circulating Cell-Free DNA Extract is a Stable Indicator for Diagnosis of Gastric Cancer. Pathol Oncol Res, 26:2621-2632. 10.1007/s12253-020-00869-1 32632900

[107] Cheng JC, Swarup N, Morselli M, Huang WL, Aziz M, Caggiano C, et al. (2024). Single-stranded pre-methylated 5mC adapters uncover the methylation profile of plasma ultrashort Single-stranded cell-free DNA. Nucleic Acids Res, 52:e50. 10.1093/nar/gkae276 PMC11194076 38797520

[108] Motea EA, Berdis AJ (2010). Terminal deoxynucleotidyl transferase: the story of a misguided DNA polymerase. Biochim Biophys Acta, 1804:1151-1166. 10.1016/j.bbapap.2009.06.030 PMC2846215 19596089

[109] Gansauge MT, Gerber T, Glocke I, Korlevic P, Lippik L, Nagel S, et al. (2017). Single-stranded DNA library preparation from highly degraded DNA using T4 DNA ligase. Nucleic Acids Res, 45:e79. 10.1093/nar/gkx033 PMC5449542 28119419

[110] Troll CJ, Kapp J, Rao V, Harkins KM, Cole C, Naughton C, et al. (2019). A ligation-based single-stranded library preparation method to analyze cell-free DNA and synthetic oligos. BMC Genomics, 20:1023. 10.1186/s12864-019-6355-0 PMC6935139 31881841

[111] Kim H, Nguyen NP, Turner K, Wu S, Gujar AD, Luebeck J, et al. (2020). Extrachromosomal DNA is associated with oncogene amplification and poor outcome across multiple cancers. Nat Genet, 52:891-897. 10.1038/s41588-020-0678-2 PMC7484012 32807987

[112] Yang L, Jia R, Ge T, Ge S, Zhuang A, Chai P, et al. (2022). Extrachromosomal circular DNA: biogenesis, structure, functions and diseases. Signal Transduct Target Ther, 7:342. 10.1038/s41392-022-01176-8 PMC9527254 36184613

[113] Ly P, Cleveland DW (2017). Rebuilding Chromosomes After Catastrophe: Emerging Mechanisms of Chromothripsis. Trends Cell Biol, 27:917-930. 10.1016/j.tcb.2017.08.005 PMC5696049 28899600

[114] Korbel JO, Campbell PJ (2013). Criteria for inference of chromothripsis in cancer genomes. Cell, 152:1226-1236. 10.1016/j.cell.2013.02.023 23498933

[115] Vogt N, Gibaud A, Lemoine F, de la Grange P, Debatisse M, Malfoy B (2014). Amplicon rearrangements during the extrachromosomal and intrachromosomal amplification process in a glioma. Nucleic Acids Res, 42:13194-13205. 10.1093/nar/gku1101 PMC4245956 25378339

[116] Gresham D, Usaite R, Germann SM, Lisby M, Botstein D, Regenberg B (2010). Adaptation to diverse nitrogen-limited environments by deletion or extrachromosomal element formation of the GAP1 locus. Proc Natl Acad Sci USA, 107:18551-18556. 10.1073/pnas.1014023107 PMC2972935 20937885

[117] Kohl NE, Kanda N, Schreck RR, Bruns G, Latt SA, Gilbert F, et al. (1983). Transposition and amplification of oncogene-related sequences in human neuroblastomas. Cell, 35:359-367. 10.1016/0092-8674(83)90169-1 6197179

[118] Karami Fath M, Karimfar N, Fazlollahpour Naghibi A, Shafa S, Ghasemi Shiran M, Ataei M, et al. (2022). Revisiting characteristics of oncogenic extrachromosomal DNA as mobile enhancers on neuroblastoma and glioma cancers. Cancer Cell Int, 22:200. 10.1186/s12935-022-02617-8 PMC9131661 35614494

[119] Wu S, Turner KM, Nguyen N, Raviram R, Erb M, Santini J, et al. (2019). Circular ecDNA promotes accessible chromatin and high oncogene expression. Nature, 575:699-703. 10.1038/s41586-019-1763-5 PMC7094777 31748743

[120] Hung KL, Yost KE, Xie L, Shi Q, Helmsauer K, Luebeck J, et al. (2021). ecDNA hubs drive cooperative intermolecular oncogene expression. Nature, 600:731-736. 10.1038/s41586-021-04116-8 PMC9126690 34819668

[121] Huh YO, Tang G, Talwalkar SS, Khoury JD, Ohanian M, Bueso-Ramos CE, et al. (2016). Double minute chromosomes in acute myeloid leukemia, myelodysplastic syndromes, and chronic myelomonocytic leukemia are associated with micronuclei, MYC or MLL amplification, and complex karyotype. Cancer Genet, 209:313-320. 10.1016/j.cancergen.2016.05.072 27318442

[122] Fan Y, Mao R, Lv H, Xu J, Yan L, Liu Y, et al. (2011). Frequency of double minute chromosomes and combined cytogenetic abnormalities and their characteristics. J Appl Genet, 52:53-59. 10.1007/s13353-010-0007-z 21107781

[123] Lv W, Pan X, Han P, Wang Z, Feng W, Xing X, et al. (2022). Circle-Seq reveals genomic and disease-specific hallmarks in urinary cell-free extrachromosomal circular DNAs. Clin Transl Med, 12:e817. 10.1002/ctm2.817 PMC9042798 35474296

[124] Zhu J, Zhang F, Du M, Zhang P, Fu S, Wang L (2017). Molecular characterization of cell-free eccDNAs in human plasma. Sci Rep, 7:10968. 10.1038/s41598-017-11368-w PMC5591271 28887493

[125] Tsai SQ, Nguyen NT, Malagon-Lopez J, Topkar VV, Aryee MJ, Joung JK (2017). CIRCLE-seq: a highly sensitive in vitro screen for genome-wide CRISPR-Cas9 nuclease off-targets. Nat Methods, 14:607-614. 10.1038/nmeth.4278 PMC5924695 28459458

[126] Mehta D, Cornet L, Hirsch-Hoffmann M, Zaidi SS-e-A, Vanderschuren H (2020). Full-length sequencing of circular DNA viruses and extrachromosomal circular DNA using CIDER-Seq. Nat Protoc, 15:1673-1689. 10.1038/s41596-020-0301-0 32246135

[127] Bao Y, Sui X, Wang X, Qu N, Xie Y, Cong Y, et al. (2024). Extrachromosomal circular DNA landscape of breast cancer with lymph node metastasis. Int J Cancer, 155:756-765. 10.1002/ijc.34985 38693790

[128] Chen Z, Qi Y, He J, Xu C, Ge Q, Zhuo W, et al. (2022). Distribution and characterization of extrachromosomal circular DNA in colorectal cancer. Mol Biomed, 3:38. 10.1186/s43556-022-00104-0 PMC9718908 36459282

[129] Ye J, Huang P, Ma K, Zhao Z, Hua T, Zai W, et al. (2023). Genome-Wide Extrachromosomal Circular DNA Profiling of Paired Hepatocellular Carcinoma and Adjacent Liver Tissues. Cancers (Basel), 15(22):5309. 10.3390/cancers15225309 PMC10670553 38001569

[130] Tan E, Liu D, Perry L, Zhu J, Cid-Serra X, Deane A, et al. (2023). Cell-free DNA as a potential biomarker for acute myocardial infarction: A systematic review and meta-analysis. Int J Cardiol Heart Vasc, 47:101246. 10.1016/j.ijcha.2023.101246 PMC10407200 37560328

[131] Zemmour H, Planer D, Magenheim J, Moss J, Neiman D, Gilon D, et al. (2018). Non-invasive detection of human cardiomyocyte death using methylation patterns of circulating DNA. Nat Commun, 9:1443. 10.1038/s41467-018-03961-y PMC5915384 29691397

[132] Liu Q, Ma J, Deng H, Huang SJ, Rao J, Xu WB, et al. (2020). Cardiac-specific methylation patterns of circulating DNA for identification of cardiomyocyte death. BMC Cardiovasc Disord, 20:310. 10.1186/s12872-020-01587-x PMC7322904 32600304

[133] Dhondup Y, Ueland T, Dahl CP, Askevold ET, Sandanger Ø, Fiane A, et al. (2016). Low Circulating Levels of Mitochondrial and High Levels of Nuclear DNA Predict Mortality in Chronic Heart Failure. J Card Fail, 22:823-828. 10.1016/j.cardfail.2016.06.013 27349571

[134] Santamaria BA, Luquin IB, Corroza J, Miguel MS, Roldan M, Zueco S, et al. (2020). Liquid biopsy shows differences in cfDNA fragmentation pattern between AD patients and controls. Alzheimer's & Dementia, 16:e039748.

[135] Nidadavolu LS, Feger D, Wu Y, Grodstein F, Gross AL, Bennett DA, et al. (2022). Circulating Cell-Free Genomic DNA Is Associated with an Increased Risk of Dementia and with Change in Cognitive and Physical Function. J Alzheimers Dis, 89:1233-1240. 10.3233/JAD-220301 PMC9969834 36031893

[136] Zhu Z, Chen T, Zhang M, Shi X, Yu P, Liu J, et al. (2025). Dynamic profiling of Cell-free DNA fragmentation uncovers postprandial metabolic and immune alterations. Hum Genomics, 19:27. 10.1186/s40246-025-00739-4 PMC11921681 40102951

[137] Lande R, Gregorio J, Facchinetti V, Chatterjee B, Wang YH, Homey B, et al. (2007). Plasmacytoid dendritic cells sense self-DNA coupled with antimicrobial peptide. Nature, 449:564-569. 10.1038/nature06116 17873860

[138] Sisirak V, Sally B, D'Agati V, Martinez-Ortiz W, Özçakar ZB, David J, et al. (2016). Digestion of Chromatin in Apoptotic Cell Microparticles Prevents Autoimmunity. Cell, 166:88-101. 10.1016/j.cell.2016.05.034 PMC5030815 27293190

[139] Duvvuri B, Lood C (2019). Cell-Free DNA as a Biomarker in Autoimmune Rheumatic Diseases. Front Immunol, 10:502. 10.3389/fimmu.2019.00502 PMC6433826 30941136

[140] Hashimoto T, Yoshida K, Hashiramoto A, Matsui K (2021). Cell-Free DNA in Rheumatoid Arthritis. Int J Mol Sci, 22. 10.3390/ijms22168941 PMC8396202 34445645

[141] Wang F, Miao HB, Pei ZH, Chen Z (2022). Serological, fragmentomic, and epigenetic characteristics of cell-free DNA in patients with lupus nephritis. Front Immunol, 13:1001690. 10.3389/fimmu.2022.1001690 PMC9791112 36578480

[142] Chan RW, Jiang P, Peng X, Tam LS, Liao GJ, Li EK, et al. (2014). Plasma DNA aberrations in systemic lupus erythematosus revealed by genomic and methylomic sequencing. Proc Natl Acad Sci USA, 111:E5302-5311. 10.1073/pnas.1421126111 PMC4267379 25427797

[143] Andersson D, Kristiansson H, Luna Santamaría M, Zafar H, Mijakovic I, Torinsson Naluai Å, et al. (2025). Evaluation of automatic cell free DNA extraction metrics using different blood collection tubes. Sci Rep, 15:19364. 10.1038/s41598-025-03508-4 PMC12134210 40461680

[144] Korabecna M, Zinkova A, Brynychova I, Chylikova B, Prikryl P, Sedova L, et al. (2020). Cell-free DNA in plasma as an essential immune system regulator. Sci Rep, 10:17478. 10.1038/s41598-020-74288-2 PMC7566599 33060738

[145] Liu Y (2022). At the dawn: cell-free DNA fragmentomics and gene regulation. Br J Cancer, 126:379-390. 10.1038/s41416-021-01635-z PMC8810841 34815523

[146] Doebley AL, Ko M, Liao H, Cruikshank AE, Santos K, Kikawa C, et al. (2022). A framework for clinical cancer subtyping from nucleosome profiling of cell-free DNA. Nat Commun, 13:7475. 10.1038/s41467-022-35076-w PMC9719521 36463275

[147] Shen H, Liu J, Chen K, Li X (2024). Language model enables end-to-end accurate detection of cancer from cell-free DNA. Brief Bioinform, 25(2):bbae053. 10.1093/bib/bbae053 PMC10883418 38385880

[148] Liu J, Shen H, Yang Y, Yang M, Zhang Q, Chen K, et al. (2024). Transformer-based representation learning and multiple-instance learning for cancer diagnosis exclusively from raw sequencing fragments of bisulfite-treated plasma cell-free DNA. Mol Oncol, 18:2755-2769. 10.1002/1878-0261.13745 PMC11547222 39380154

[149] Sangphukieo A, Noisagul P, Thongkumkoon P, Chaiyawat P. 2024. Ultra-low coverage fragmentomic model of cell-free DNA for cancer detection based on whole-exome regions. ELife. 13:RP95320.

[150] Ulz P, Perakis S, Zhou Q, Moser T, Belic J, Lazzeri I, et al. (2019). Inference of transcription factor binding from cell-free DNA enables tumor subtype prediction and early detection. Nat Commun, 10:4666. 10.1038/s41467-019-12714-4 PMC6789008 31604930

[151] Liu MC, Oxnard GR, Klein EA, Swanton C, Seiden MV (2020). Sensitive and specific multi-cancer detection and localization using methylation signatures in cell-free DNA. Ann Oncol, 31:745-759. 10.1016/j.annonc.2020.02.011 PMC8274402 33506766

[152] Siravegna G, Marsoni S, Siena S, Bardelli A (2017). Integrating liquid biopsies into the management of cancer. Nat Rev Clin Oncol, 14:531-548. 10.1038/nrclinonc.2017.14 28252003

[153] Kansal AR, Tafazzoli A, Shaul A, Chavan A, Ye W, Zou D, et al. (2024). Cost-effectiveness of a multicancer early detection test in the US. Am J Manag Care, 30:e352-e358. 10.37765/ajmc.2024.89643 39745501

[154] Merker JD, Oxnard GR, Compton C, Diehn M, Hurley P, Lazar AJ, et al. (2018). Circulating Tumor DNA Analysis in Patients With Cancer: American Society of Clinical Oncology and College of American Pathologists Joint Review. J Clin Oncol, 36:1631-1641. 10.1200/JCO.2017.76.8671 29504847

[155] Bryzgunova OE, Laktionov PP (2015). Extracellular Nucleic Acids in Urine: Sources, Structure, Diagnostic Potential. Acta Naturae, 7:48-54. PMC4610164 26483959

[156] Qi T, Pan M, Shi H, Wang L, Bai Y, Ge Q (2023). Cell-Free DNA Fragmentomics: The Novel Promising Biomarker. Int J Mol Sci, 24:1503. 10.3390/ijms24021503 PMC9866579 36675018

[157] Wang G, Christensen LA, Vasquez KM (2006). Z-DNA-forming sequences generate large-scale deletions in mammalian cells. Proc Natl Acad Sci USA, 103:2677-2682. 10.1073/pnas.0511084103 PMC1413824 16473937

[158] Panda A, Alahi I, Chauhan PS, Sheng J, Colon N, Nawaf C, et al. (2024). Abstract 965: Urine cell-free DNA multi-omics combining copy number analysis with fragmentomics to detect minimal residual disease in bladder cancer patients. Cancer Research, 84:965-965.

[159] Medina JE, Annapragada AV, Lof P, Short S, Bartolomucci AL, Mathios D, et al. (2025). Early Detection of Ovarian Cancer Using Cell-Free DNA Fragmentomes and Protein Biomarkers. Cancer Discov, 15:105-118. 10.1158/2159-8290.CD-24-0393 PMC11726017 39345137

